# Supplementary material for: Causal Relationship between Aging and Anorexia Nervosa: A White-Matter-Microstructure-Mediated Mendelian Randomization Analysis
Source: Biomedicines. 2024 Aug 16;12(8):1874. doi: 10.3390/biomedicines12081874 (PMC11351342; doi:10.3390/biomedicines12081874)
Supplement: Supplementary file 1 [file biomedicines-12-01874-s001.zip › Supplementary Figure/Figure S4/Singe SNP plot of MR analysis for TL to ubm-b-1496.pdf]

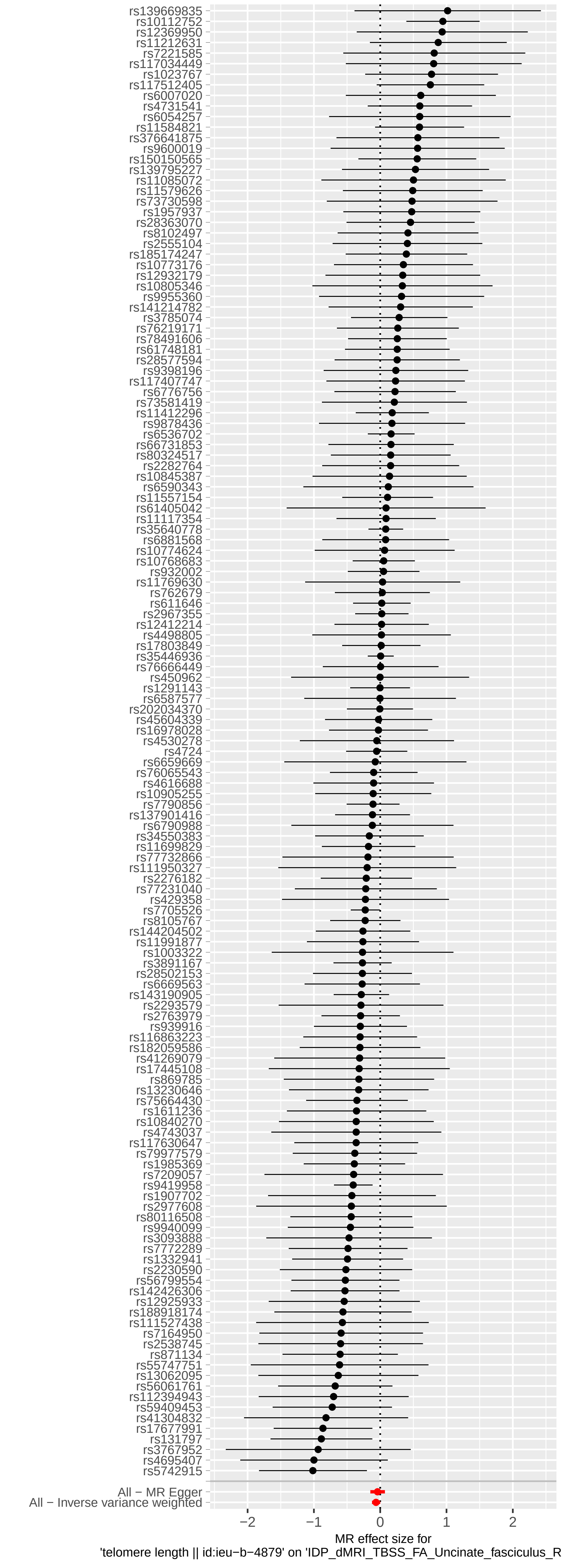
rs139669835
rs10112752
rs12369950
rs11212631
rs7221585
rs117034449
rs1023767
rs117512405
rs6007020
rs4731541
rs6054257
rs11584821
rs376641875
rs9600019
rs150150565
rs139795227
rs11085072
rs11579626
rs73730598
rs1957937
rs28363070
rs8102497
rs2555104
rs185174247
rs10773176
rs12932179
rs10805346
rs9955360
rs141214782
rs3785074
rs76219171
rs78491606
rs61748181
rs28577594
rs9398196
rs117407747
rs6776756
rs73581419
rs11412296
rs9878436
rs6536702
rs66731853
rs80324517
rs2282764
rs10845387
rs6590343
rs11557154
rs61405042
rs11117354
rs35640778
rs6881568
rs10774624
rs10768683
rs932002
rs11769630
rs762679
rs611646
rs2967355
rs12412214
rs4498805
rs17803849
rs35446936
rs76666449
rs450962
rs1291143
rs6587577
rs202034370
rs45604339
rs16978028
rs4530278
rs4724
rs6659669
rs76065543
rs4616688
rs10905255
rs7790856
rs137901416
rs6790988
rs34550383
rs11699829
rs77732866
rs111950327
rs2276182
rs77231040
rs429358
rs7705526
rs8105767
rs144204502
rs11991877
rs1003322
rs3891167
rs28502153
rs6669563
rs143190905
rs2293579
rs2763979
rs939916
rs116863223
rs182059586
rs41269079
rs17445108
rs869785
rs13230646
rs75664430
rs1611236
rs10840270
rs4743037
rs117630647
rs79977579
rs1985369
rs7209057
rs9419958
rs1907702
rs2977608
rs80116508
rs9940099
rs3093888
rs7772289
rs1332941
rs2230590
rs56799554
rs142426306
rs12925933
rs188918174
rs111527438
rs7164950
rs2538745
rs871134
rs55747751
rs13062095
rs56061761
rs112394943
rs59409453
rs41304832
rs17677991
rs131797
rs3767952
rs4695407
rs5742915
All − MR Egger
All − Inverse variance weighted
−2
−1
0
1
2
MR effect size for
'telomere length || id:ieu−b−4879' on 'IDP_dMRI_TBSS_FA_Uncinate_fasciculus_R
